# Individualized psychotherapy reveals a very high prevalence of adverse life events in functional neurological disorders

**DOI:** 10.3389/fpsyt.2025.1605028

**Published:** 2025-10-20

**Authors:** Luana Gilio, Giovanni Galifi, Marco Lipera, Barbara Aramini, Carla Antonucci, Ilaria Simonelli, Cinzia Femiano, Simone Cappellano, Giordana Pelone, Carmelo Licitra Rosa, Diego Centonze, Mario Stampanoni Bassi

**Affiliations:** ^1^ Unit of Neurology, IRCCS Neuromed, Pozzilli, IS, Italy; ^2^ Faculty of Psychology, Uninettuno Telematic International University, Rome, Italy; ^3^ Department of Systems Medicine, Tor Vergata University, Rome, Italy; ^4^ Istituto Italiano di Psicoanalisi per la Ricerca e la Clinica PSICOMED, Pozzilli, IS, Italy; ^5^ Biostatistics Service, CTC, Fatebenefratelli Gemelli-Isola, Rome, Italy; ^6^ Unit of Neuromotor Rehabilitation, IRCCS San Raffaele Pisana, Rome, Italy

**Keywords:** functional neurological disorder, psychotherapy, psychoanalysis, adverse life events, standardized evaluation, trauma

## Abstract

**Background:**

Adverse life events are frequent in patients with functional neurological disorder (FND), although their prevalence is highly variable when assessed using standardized methods. We explored whether an extended multimodal evaluation with a personalized approach may yield additional insights.

**Methods:**

This cross-sectional study included 83 newly diagnosed FND patients and 82 organic neurological disorder (OND) patients. All participants underwent: (1) a standardized psychological/psychometric evaluation of mood, fatigue, sleep, personality disorders. Adverse life events were specifically investigated using the Life Stressor Checklist-R (LSCL-R); (2) a brief psychotherapeutic intervention consisting in five-eight sessions of psychodynamic psychotherapy. We compared the prevalence of adverse life events in the two evaluations.

**Results:**

Increased prevalence of adverse life events was found in FND compared with OND patients using both the LSCL-R (66.3% vs 36.6%) and the brief psychotherapeutic intervention (87.9% vs 45.1%). While in the OND group the two evaluations demonstrated a significant agreement, the brief psychotherapeutic intervention showed traumatic events in a consistent proportion of FND patients reporting no adverse life experiences in the LSCL-R. Traumatic events evaluated using both the LSCL-R and the brief psychotherapeutic intervention were significantly associated with FND group controlling for other clinical characteristics (OR=3.625, 95%CI 1.812–7.250, p<0.001; OR=10.731, 95%CI 4.417–26.071, p<0.001, respectively).

**Conclusions:**

A brief psychotherapeutic intervention uncovered a high prevalence of adverse life events in patients with FND, suggesting that a significant number of traumatic experiences remain undetected during standard evaluations. These findings have important implications for the pathogenesis and treatment of FND, and support the inclusion of psychotherapeutic assessment as part of a multidisciplinary approach.

## 1 Introduction

Functional neurological disorders (FND) represent a frequent condition associated with significant disability and quality of life deterioration (1). In recent years, renewed interest in FND has coincided with a better clinical definition and the adoption of shared diagnostic criteria. Nevertheless, unresolved problems regarding the pathogenesis and the treatment of FND still exist.

The presence of adverse stressful experiences and traumatic events preceding the onset of symptoms has traditionally represented a key requirement for the diagnosis of FND (2). However, previous studies have shown that, although the prevalence of remote or recent adverse life events is higher among individuals with FND, a significant proportion of patients report no stressful events (3). According to the Diagnostic and Statistical Manual of Mental Disorders, Fifth Edition (DSM-5), the presence of traumatic events or psychological stressors is no longer required for the diagnosis of FND, which is now based on both the exclusion of organic disease and on the presence of positive clinical signs and symptoms (4, 5). While the value of a neutral terminology is not in doubt, clarifying the real incidence of traumatic events in patients with FND may be important to better understand the pathogenesis of this condition and design targeted therapies.

Critical issues influencing the assessment of past adverse life experiences are the patient’s ability to recognize and actively report such events, and more importantly, the actual definition of what may constitute a predisposing or triggering traumatic event (6). In this regard, the highly individualized approach of psychoanalysis may be useful to clarify the real incidence of factors relevant to the patient and fill the gap left by the standardized scales and questionnaires employed in clinical practice (7). Psychoanalytically oriented psychotherapy, through its focus on in-depth exploration of the patient’s unconscious processes, provides a means to uncover memories, feelings and conflicts that may not be easily accessible through more structured assessments (8). This approach can complement or even improve the results of standardized questionnaires, promoting increased awareness of previous adverse life experiences (9, 10). Psychodynamic psychotherapy provides a robust framework for understanding how trauma in individual, familial, and sociocultural contexts shapes the development and maintenance of symptoms (11, 12).

In this cross-sectional study, we evaluated in a group of patients diagnosed with FND the prevalence of remote and recent adverse life events, assessed by a standardized psychological/psychometric evaluation and by a brief (5 to 8 sessions) psychotherapeutic intervention based on a psychodynamic approach. The results were compared with a group of patients diagnosed with organic neurological disorder (OND) and without functional symptoms.

## 2 Methods

### 2.1 Study population and study design

We conducted a single-center cross-sectional study including patients referred to the Neurology unit of IRCCS Neuromed Hospital (IS), Italy, between 2020 and 2022. The study was approved by the Ethics Committee of IRCCS Neuromed (CE number 04/20) and conducted according to the Declaration of Helsinki. All participants provided written informed consent.

A group of consecutive patients with a new diagnosis of FND (functional motor, sensory or cognitive symptoms, functional seizures, or mixed symptoms) were included. The diagnosis of FND was based on the DSM-5 Criteria (F44.4-7). The diagnosis was established by a neurologist using positive signs and symptoms. Additional instrumental examination was prescribed if requested to formulate the diagnosis.

We also enrolled a consecutive group of patients with a confirmed diagnosis of organic CNS disorders or peripheral nervous system disorders, without functional neurological symptoms, referred to the psychotherapy service.

Exclusion criteria were: (i) a neurodegenerative CNS disease (ii) severe psychiatric conditions (acute suicidality, active psychotic symptoms); (iii) alcohol or drug abuse; and (iv) insufficient language skills.

Demographic characteristics, including sex and age, were recorded at the time of enrollment. In FND patients, clinical characteristics were also recorded (age at symptoms onset, duration of symptoms, type of FND).

### 2.2 Psychological/psychometric evaluation

Psychological assessment was performed by a trained psychologist blind to the diagnosis. The interview was carried out in approximately 1.5h.

The presence of depression was assessed using the Beck Depression Inventory - Second Edition (BDI-II) (13, 14). Anxiety levels were assessed using the State-Trait Anxiety Inventory - Form Y (STAI-Y) (15). The 19-item Pittsburgh Sleep Quality Index (PSQI) was used to measure subjective experience of sleep quality in the previous month (16). Fatigue severity was assessed using the Fatigue Severity Scale (FSS) (17).

Personality disorders (PD) were analyzed with the Structured Clinical Interview for DSM-5 Personality Disorders (SCID-5-PD) (18).

The Life Stressor Checklist-R (LSCL-R) was used for assessing stressful or traumatic life events (19, 20).

Traumatic events and adverse stressful experiences are major etiological factors in a wide variety of physical and mental disorders (21).

The LSCL-R is a self-report questionnaire that includes 30 adverse life events, such as experiences with natural disasters, physical or sexual assault, death of a relative, illness and other events also including features open-ended items allowing respondents to report any event not explicitly addressed by the predefined items on the questionnaire, following a yes/no response format. For endorsed events, participants were required to specify the age at which the event occurred (19, 20).

Traumatic events were classified as in Reuber et al., 2007 (22): sexual trauma, non-sexual trauma (including childhood physical or emotional abuse, witnessing domestic violence in childhood, being bullied as a child, being an adult victim of domestic violence, being a victim of other assaults, involvement in accidents, workplace bullying), bereavements (only included if the patient showed affect during the interview or considered the bereavement to have had a significant emotional impact), social/family factors (including perceived peer pressures, family or relationship difficulties, family dysfunction, health issues or breakdown, lack of social support and financial issues considered by the patient to have had a significant impact on his or her life) and personal health issues (22). Traumatic events were also classified as recent if they occurred within the last year before symptom onset, or remote if earlier.

### 2.3 Psychodynamic psychotherapy

The brief psychotherapeutic intervention, based on a psychodynamic approach, explored both remote and recent experiences relevant to the patient and their relationship to current life events. Sessions focused on the recollection and reconstruction of personal history related to present symptoms in a synchronic and diachronic perspective (8). In some cases, patients had already hypothesized a link between a previous adverse event and symptom onset, more often, such associations were missing, as symptoms may be related to childhood, adolescent, or adult experiences that have been removed. The ultimate goal was to recognize unconscious phenomena, such as conflict and adverse or traumatic experiences, and make them conscious.

Although this intervention is not standardized, every effort was made to ensure consistency across the study (23). Patients were randomly assigned to one of three psychoanalysts with consolidated experience in psychodynamic psychotherapy. Each patient always met with the same psychotherapist, and five to eight individual encounters were conducted. The number of sessions was adjusted according to patient and therapist availability and the needs of the individual case. Psychotherapists were blinded to the diagnostic category and unaware of the results of the psychological/psychometric evaluation. Regular intervision meetings were held to maintain consistency in technique, interview structure, and trauma coding.

Adverse life events were assessed through qualitative methodologies, including content analysis of the patient’s narrative and in-depth exploration of the emotional salience of reported experiences. After the brief psychotherapeutic intervention, psychotherapists classified relevant life events by type, as in Reuber et al., 2007, and by time period (recent or remote) consistent with the psychological/psychometric evaluation (22). At the end of the intervention, patients were referred for continued psychotherapeutic care to ensure long-term follow-up.

### 2.4 Statistical analysis and sample size calculation

Based on previous studies, the prevalence of traumatic events in FND patients and OND is difficult to estimate accurately and varies widely depending on the type of patients and the method used. Recent meta-analyses suggest that the frequency of traumatic events in patients is at least twice as high as in controls (3, 24). For sample size calculation, we assumed a frequency of 40% in patients with FND and 20% in OND. Considering a type I error as α = 0.05, a sample size of 162 subjects (81 cases and 81 controls) would result in a power of 80%. We conservatively selected a relatively low frequency of adverse events in FND patients and controls (40% and 20% respectively); of note, assuming higher frequencies with a similar ratio (e.g., 60% and 30%), the required sample size would be smaller.

Kolmogorov–Smirnov test was applied to verify the normality distribution of continuous variables. Continuous data were presented as median (interquartile range, IQR=25th–75th percentile). Categorical or dichotomous variables were presented in terms of frequency (percentage, %). Nonparametric Mann-Whitney test was used to compare continuous variables between FND patients and OND. Pearson’s Chi-square was used to assess differences between FND patients and OND patients in categorical variables. To assess the association between group (FND patients and OND patients) and traumatic events, adjusting for other clinical variables logistic regression was used. Bar graph was used to depict differences in the prevalence of adverse life events as assessed by the psychological/psychometric evaluation and brief psychotherapeutic intervention. To compare the results of the two evaluations, overall percent agreement (OPA), positive percent agreement (PPA) and negative percent agreement (NPA) were calculated. To address whether the difference between assessment methods varied by diagnostic group, we fitted a mixed logistic regression model including a method-by-group interaction term. A p value < 0.05 was considered statistically significant. All the analyses were performed with SPSS Statistics for Windows (IBM Corp., Armonk, NY, USA) and R.

## 3 Results

### 3.1 Demographic and clinical characteristics of FND and control patients

A total of 99 FND patients were screened (in 6 patients the diagnosis of FND was not confirmed, 10 patients were excluded as they consented to participate but subsequently dropped out). Data from 83 newly diagnosed FND patients were included in this study. The FND group included different FND subtypes: motor, N=37 (44.6%); sensory, N=19 (22.9%); functional seizures, N=9 (10.84%); cognitive, N=1 (1.2%); and 17 patients (20.5%) presented with more than one symptom type. In 13 FND patients (15.7%) a concomitant neurological disease was present: migraine, N=5; epilepsy, N=3; multiple sclerosis, N=3; spinal disc herniation, N=2).

A group of consecutive 82 patients with OND was also included (94 patients were enrolled, 12 patients were excluded due to drop out). The group included patients diagnosed with: brain vascular disease, N=15; multiple sclerosis, N=42; normal pressure hydrocephalus, N=3; pseudotumor cerebri, N=7; migraine, N=3; spondylotic myelopathy, N=8; polyneuropathy, N=4.

The study cohort included a total of 165 FND patients and OND patients, the demographic and clinical characteristics of are shown in **Table 1**.

**Table 1:** Demographic and clinical characteristics of FND and OND patients.

		FND	OND	p
Patients	N	83	82	–
Sex, F	N (%)	60 (72.3)	52 (63.4)	0.222^§^
Age	Median (IQR)	36.5 (23.9 – 46.9)	34.2 (26.7 – 45.3)	0.802
Duration of symptoms, months	Median (IQR)	14.4 (7.4 – 39.9)	–	–
BDI-II	Median (IQR)	16 (11 - 25)	14 (9.7 - 20)	0.068
Depression, yes	N (%)	53 (63.9)	50 (61)	0.703^§^
STAI-Y state/trait	Median (IQR)	41 (33 – 55)/48 (39 - 60)	46 (39 – 57)/46.5 (38 – 53)	0.072/0.149
Anxiety state/trait, yes	N (%)	49 (59)/60 (72.3)	57 (69.5)/58 (70.7)	0.160/0.825^§^
FSS	Median (IQR)	5 (3 – 5.9)	3.4 (2.1 – 4.6)	**0.001**
Fatigue, yes	N (%)	57 (68.7)	34 (41.5)	**<0.001^§^**
PSQI	Median (IQR)	7 (5 - 12)	5 (3 – 9)	**<0.001**
Sleep disturbances, yes	N (%)	65 (78.3)	48 (58.5)	**0.006^§^**
Personality, SCID-5-PD	N (%)	8 (9.6) *	3 (3.7) *	0.124^§^

**^#^** Mann-Whitney p; ^§^Chi-Square p. BDI-II, Beck Depression Inventory, Second Edition; FND, Functional Neurological Disorder; FSS, Fatigue Severity Scale; PSQI, Pittsburgh Sleep Quality Index; SCID-5-PD, Structured Clinical Interview for DSM-5 Personality Disorders; STAI-Y, State-Trait Anxiety Inventory, Form Y. *missing data: SCID5-PD in 5 FND patients (6%) and in 8 control patients (9.8%).

Significant values are in bold.

No significant differences were observed between FND and OND patients in sex distribution (p = 0.222) and age (p = 0.802). Systematic psychological/psychometric evaluation showed comparable levels of depression and anxiety between the two groups. Conversely, in FND patients, higher prevalence of fatigue (FND=68.7%, OND=41.4%, Chi square p < 0.001) and sleep disorders (FND=78.3%, OND=58.5%, Chi square p = 0.006) was found.

### 3.2 Evaluation of adverse life events using the LSCL-R

**Figure 1:**
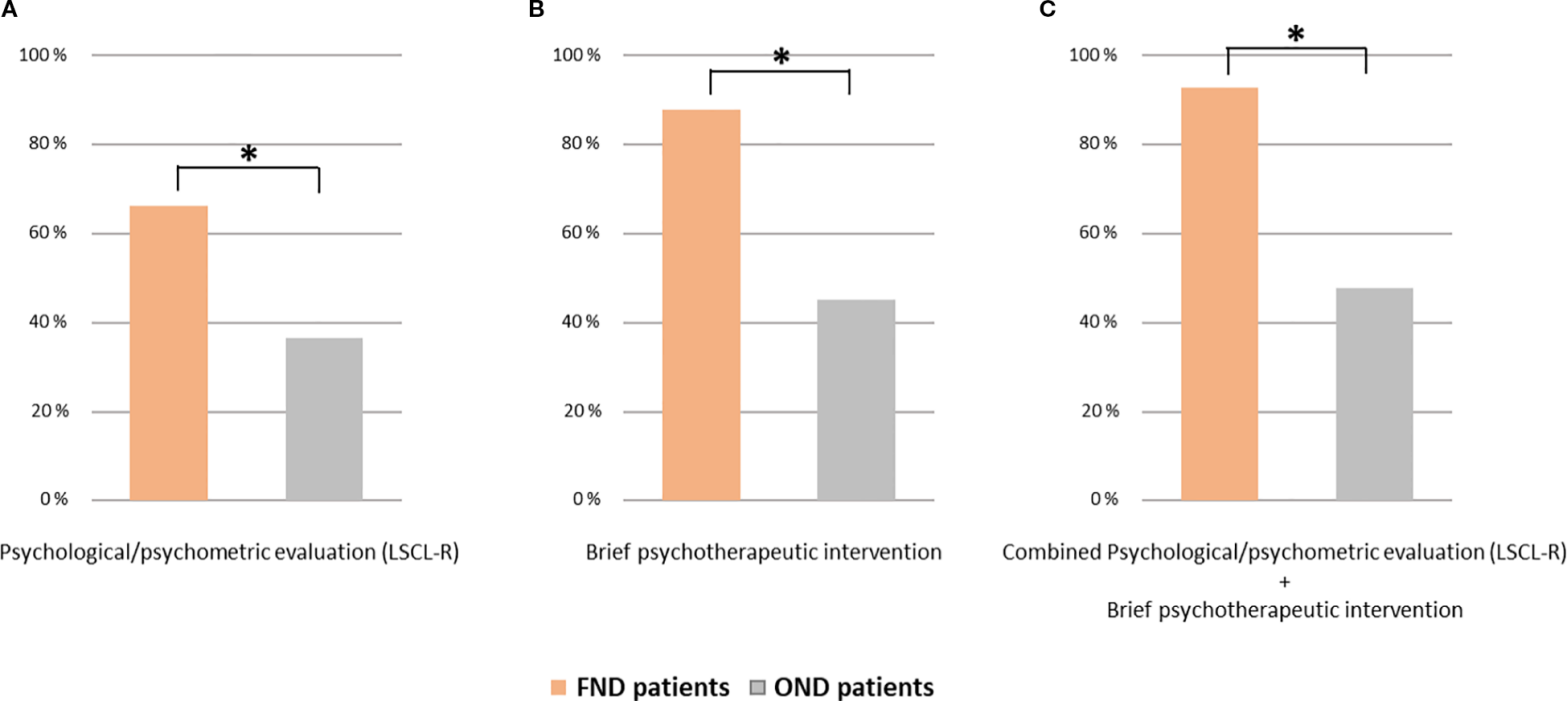
The prevalence of adverse life events in patients with FND and OND as assessed during the psychological/psychometric evaluation using the LSCL-R **(A)**, and by brief psychotherapeutic intervention **(B)**. The prevalence of adverse life events in at least one of the two assessments is also reported **(C)**. *Chi square p < 0.001. FND, functional neurological disorders; OND, organic neurological disorder; LSCL-R, Life Stressor Checklist-R.

**Figure 2:**
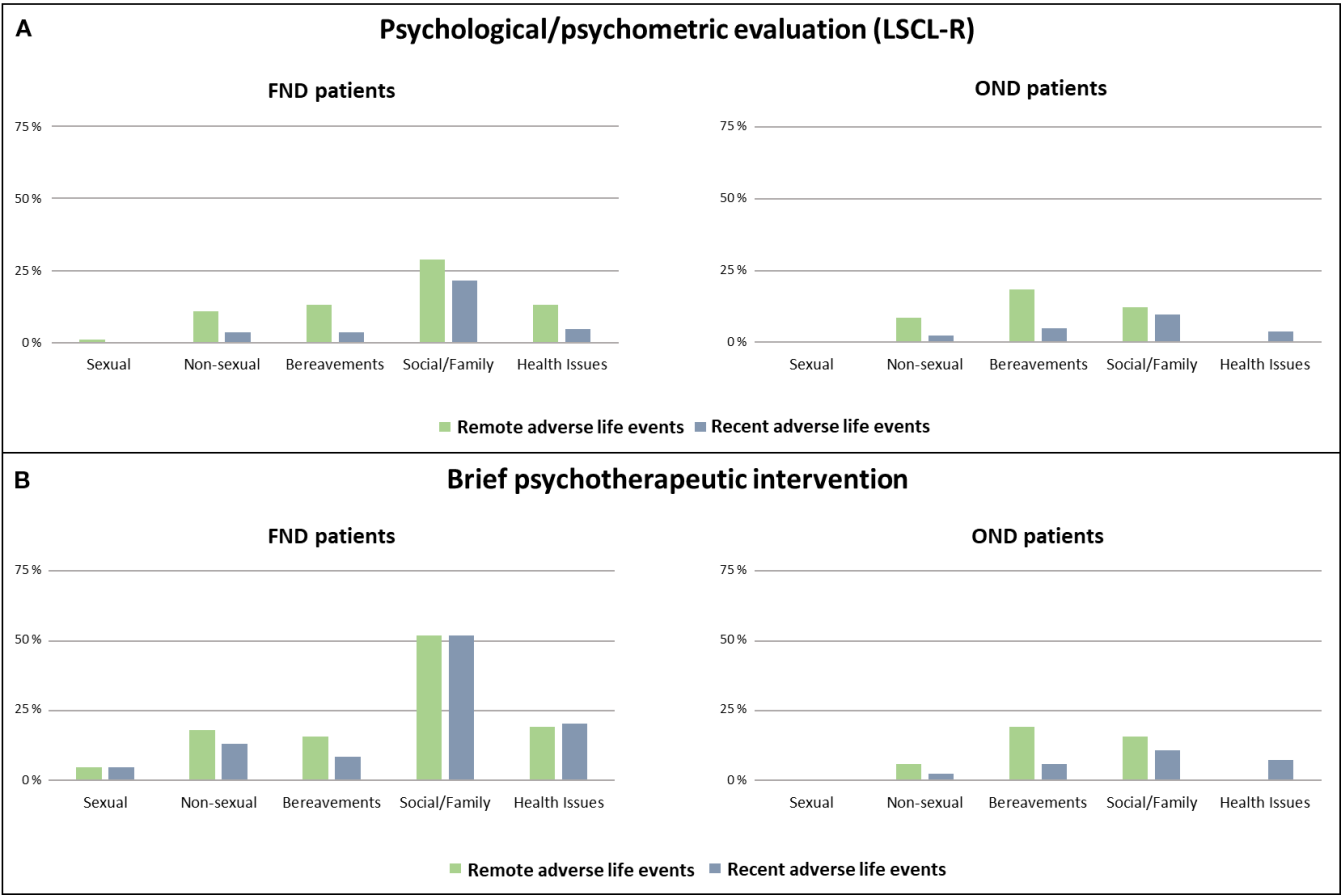
Prevalence and type of remote and recent adverse life events. Prevalence and type of remote and recent adverse life events reported by FND and OND patients during the psychological/psychometric evaluation **(A)** and the brief psychotherapeutic intervention **(B)**. Patients with FND and OND may present multiple adverse life events. FND, functional neurological disorders; OND, organic neurological disorder; LSCL-R, Life Stressor Checklist-R.

During the psychological/psychometric evaluation, using the LSCL-R, adverse life events were identified in 55 (66.3%) of FND patients and in 30 (36.6%) OND patients (Chi-Square p < 0.001) (**Figure 1A**). Both remote and recent adverse life events were more frequently observed in FND compared to OND patients. Remote adverse events were reported by 45 (54.2%) FND patients and 19 (23.2%) OND patients (Chi-Square p < 0.001). A recent adverse event was found in 32 (38.6%) of FND patients and in 14 (17.1%) of OND patients (Chi-Square p = 0.002). The type of adverse life event is shown in **Figure 2A**.

Notably, in 28 patients with FND (33.7%) no adverse life events were identified.

### 3.3 Evaluation of adverse life events by the brief psychotherapeutic intervention

At the end of the psychotherapeutic intervention [median number of sessions = 7 (IQR: 5 – 8)], for each patient the presence and the type of remote/recent adverse life events were recorded and classified ad in Reuber et al., 2007 (22). The number of sessions and the number of drop-out patients did not differ in the two groups. Adverse events were reported by 73 (87.9%) FND patients and by 37 (45.1%) OND patients (Chi-Square p < 0.001) (**Figure 1B**). Both remote and recent adverse life events were more frequently observed in FND compared to OND patients. Remote traumatic events were reported by 59 (71.1%) of FND patients, and by 27 (32.9%) of control patients (Chi-Square p < 0.001). Recent traumatic experiences were found in 60 (72.3%) of FND patients and in 19 (23.2%) OND patients (Chi-Square p < 0.001). The type of adverse life event assessed by the brief psychotherapeutic intervention is shown in **Figure 2B**.

Notably, combining the LSCL-R and the brief psychotherapeutic intervention, an adverse life event was observed in 77 (92.8%) FND patients and in 39 (47.6%) OND patients (Chi-Square p < 0.001) (**Figure 1C**). Only six FND patients did not report adverse life events in either of the two assessments. Their clinical characteristics were as follows: sex, female = 3/6; age, median (IQR) = 41.7 years (17.1–57.6); phenotype: 4 functional motor disorders, 1 PNES, and 1 mixed phenotype; psychiatric comorbidities = 1/6 anxiety panic attack. Due to the very small sample size, no statistical analysis was performed.

### 3.4 Association between adverse life events and FND diagnosis

To explore the association between group and presence of adverse life events in the two evaluations controlling for other clinical characteristics logistic regressions were used (**Table 2**).

**Table 2:** Logistic regressions.

Variable	Model 1			Model 2		
	OR	CI	p	OR	CI	p
Adverse life events (LSCL-R)	3.625	1.812 – 7.250	**< 0.001**	–	–	–
Adverse life events (brief psychotherapeutic intervention),	–	–	–	10.731	4.417 – 26.071	**< 0.001**
Age	1.002	0.976 – 1.028	0.978	1.016	0.987 – 1.045	0.281
Sex, F	1.109	0.527 – 2.332	0.785	0.888	0.397 – 1.984	0.772
Mood, presence	0.475	0.189 – 1.189	0.112	0.491	0.185 – 1.302	0.153
Fatigue, yes	3.077	1.549 – 6.114	**0.001**	3.395	1.611 – 7.154	**0.001**

Association between the presence of adverse life events and group (FND vs OND). Logistic regression controlling for the effect of other clinical characteristics: age, sex, presence of mood disturbances (either depression, state, or trait anxiety) and fatigue.

Significant values are in bold.

LSCL-R, Life Stressor Checklist-R.

A significant association was found between the presence of adverse life events assessed with the LSCL-R and group (FND vs OND) controlling for the effect of other clinical characteristics: age, sex, presence of mood disturbances (either depression, state, or trait anxiety) and fatigue (OR=3.625, 95%CI 1.812 – 7.250, p < 0.001). Logistic regression also showed a significant association between the presence of adverse life events assessed during the brief psychotherapeutic intervention and FND group (OR=10.731, 95%CI 4.417 – 26.071, p < 0.001).

The logistic regression analyses showed that the presence of adverse life events assessed both with LSCL-R and the brief psychotherapeutic intervention are significant predictors for FND group membership. Comparing the coefficients, the brief psychotherapeutic intervention showed a slightly increased predictive power. In addition, in both models, fatigue showed a significant association with FND group (OR=3.077, 95%CI 1.549 – 6.114, p = 0.001; and OR=3.395, 95%CI 1.611 – 7.154, p = 0.002, respectively). Comparisons between FND subgroups were not performed because of markedly different sample sizes.

Finally, we tested for a potential interaction between assessment method and diagnostic group using a mixed logistic regression model, given the binary nature of the outcome. The method-by-group interaction was statistically significant (p = 0.037), indicating that the probability of detecting a traumatic event differs between groups depending on the assessment method. In the FND group, the probability of detecting adverse events was 86% (95% CI: 78%–95%) with the brief psychotherapeutic intervention and 68% (95% CI: 60%–77%) with the LSCL-R. In the OND group, the corresponding probabilities were 44% (95% CI: 32%–55%) and 35% (95% CI: 25%–44%), respectively.

### 3.5 Comparison between the LSCL-R and the brief psychotherapeutic intervention

We compared the results of the LSCL-R, administered during the psychological/psychometric evaluation, and of the brief psychotherapeutic intervention in the two groups.

In the OND group, the two evaluations showed a substantial agreement (Kappa = 0.724, p < 0.001), indicating a significant concordance in the assessment of adverse events. The prevalence of adverse events was slightly increased when assessed with the brief psychotherapeutic intervention (45.1 vs 36.6%), although the difference was not statistically significant (McNemar p = 0.065). As shown in **Table 3**, in the OND group the brief psychotherapeutic intervention and the LSCL-R showed an 86.6% overall percent agreement (OPA), a positive percent agreement (PPA) of 93.3%, and a negative percent agreement (NPA) of 82.7%.

**Table 3:** Adverse life events: psychological/psychometric evaluation and brief psychotherapeutic intervention.

OND group				
		Brief psychotherapeutic intervention		OPA= 86.6%
		Yes (N=37)	No (N=45)	
Psychological/psychometric evaluation (LSCL-R)	Yes (N=30)	28	2	PPA= 93.3%
	No (N=52)	9	43	NPA= 82.7%
FND group				
		Brief psychotherapeutic intervention		OPA= 68.7%
		Yes (N=73)	No (N=10)	
Psychological/psychometric evaluation (LSCL-R)	Yes (N=55)	51	4	PPA= 92.7%
	No (N=28)	22	6	NPA= 21.4%

To compare the presence of adverse life events assessed during the psychological/psychometric evaluation and the brief psychotherapeutic intervention, the overall percent agreement (OPA), positive percent agreement (PPA) and negative percent agreement (NPA) were calculated.

FND, functional neurological disorders; LSCL-R, Life Stressor Checklist-R; OND, organic neurological diseases; NPA, negative percent agreement; OPA, overall percent agreement; PPA, positive percent agreement.

In FND patients, the brief psychotherapeutic intervention showed a significantly higher prevalence of adverse events compared with the LSCL-R (87.9% vs 66.3%, McNemar p = 0.001). The agreement between the two evaluations was non-significant (Kappa = 0.168, p = 0.061). The brief psychotherapeutic intervention and the psychological/psychometric assessment showed a 68.7% OPA. PPA was 92.7%, indicating that in patients showing adverse events at the LSCL-R, the brief psychotherapeutic intervention confirmed the finding in a very high proportion of cases. Conversely, the NPA was only 21.4%. Accordingly, among 28 patients reporting no adverse life experiences in the LSCL-R, the brief psychotherapeutic intervention identified a significant traumatic event in 22 patients (78.5%) (**Table 3**).

To better analyze this finding, we have further detailed in these 22 patients the type of traumatic event identified during the brief psychotherapeutic intervention. A remote adverse life event was found in 16 patients, a recent adverse life event in 18 patients, and 12 patients reported both remote and recent adverse life events. These events were classified as social/family (N=22), health issues (N=12), bereavement (N=7), nonsexual (N=6). 9 of these patients presented more than one type of trauma. In 3 cases, the events were extremely private, and shame or resistance to share highly confidential information may have played a role. In 7 cases, patients spontaneously reported during psychotherapy personal traumatic experiences, although the role of these events was previously minimized or denied by the patient. In 6 cases, significant events highly relevant to personal history emerged and were identified as traumatic during psychotherapy. Finally, in 6 patients no evident reasons were identified.

## 4 Discussion

In patients with FND, after the confirmed diagnosis, a comprehensive psychological/psychometric evaluation is routinely performed to assess the presence of predisposing, precipitating and perpetuating factors included in the biopsychosocial model (25, 26). Mood and personality disorders, sleep disturbances and fatigue, are frequently observed in patients with FND and have been associated with worse disease course and poor response to treatments (22, 25–34). Moreover, among psychological and social factors, remote and recent adverse life events have received particular attention and represent established predisposing and precipitating conditions for FND (25, 32, 35).

The method used to assess traumatic events may play an important role in estimating their prevalence, as evidenced by the considerable variability in previous studies in FND patients (3). Although difficult to identify at the time of diagnosis, remote and recent adverse life experiences are more frequent in patients with FND than in control patients when assessed using standardized questionnaires (3). Using the same approach, during the psychological/psychometric evaluation, we found a higher prevalence of remote and recent adverse life experiences in the FND group compared with OND patients. Notably, during this assessment almost one-third of FND patients reported no stressful or traumatic factors as assessed by the LSCL-R. These findings are in line with a recent meta-analysis showing that a significant proportion (14 – 68%) of patients with FND do not report remote or recent adverse life events when assessed with standardized questionnaires (3, 24).

The psychological/psychometric evaluation also showed that the prevalence of depression and anxiety was high in both groups, although comparable between FND patients and our group of patients with OND referred to psychotherapy. Conversely, increased prevalence of fatigue and sleep disturbances were found in the FND group. These findings are in line with previous literature and confirm the importance of systematically screening patients with FND for potentially treatable associated conditions (27–30).In the present study we explored whether and extended evaluation with a personalized approach can be helpful in assessing the incidence of remote or recent adverse life experiences. After the conventional psychological/psychometric assessment, all participants underwent a short course (median number of sessions = 6) of psychotherapy based on a psychodynamic approach. This brief psychotherapeutic intervention confirmed a higher prevalence of adverse events in FND patients comparing with OND patients. In the FND group the prevalence of adverse life events reported during the brief psychotherapeutic intervention was significantly higher than evidenced during the psychological/psychometric evaluation. Notably, in a significant proportion of FND patients who did not report remote or recent adverse life experiences in the LSCL-R, adverse events were identified during the brief psychotherapeutic intervention. Conversely, in patients reporting adverse events at the LSCL-R the brief psychotherapeutic intervention confirmed the finding in > 90% of cases. Finally, in the OND group, we found a substantial agreement between the two evaluations, with a slight increase in the number of adverse events identified after the brief psychotherapeutic intervention that is not significant.

The results of the personalized psychotherapeutic intervention suggest that a significant number of remote and recent life traumatic events may not be detected during conventional psychological/psychometric evaluations.

Regarding the higher frequency of traumatic events identified during the brief psychotherapeutic intervention, some considerations can be drawn. The limited time available for interviews and for establishing a trust relationship with the examiner can significantly reduce the patient’s ability and willingness to share personal information and to recall remote episodes. Indeed, the brief psychotherapeutic intervention may enhance the detection of adverse life events by providing more time for interviews, with a median duration of about 7 hours, which is considerably longer than in most previous studies. Furthermore, repeated meetings with the same psychotherapist over a two-month period fostered the development of a trusting relationship, which is often essential for patients to disclose sensitive information (36). Nevertheless, other mechanisms may also have contributed to the higher detection rate of traumatic events, including a greater tendency to report such experiences due to disclosure bias. Future studies should assess the level of patient engagement with the intervention (e.g., resistance, perceived benefits) to better clarify these dynamics.

An important aspect concerns the patient’s ability to recognize and report an event as traumatic. As previously suggested, to be detected by a systematic assessment, relevant events must have been recognized as traumatic and then actively reported (6). This highlights the value of combining standardized questionnaires with individualized methods aimed at assessing a more subjective definition of trauma (6). Notably, in our study, several events reported as traumatic were experiences that might appear irrelevant to most people and would likely remain undetected by conventional instruments. In some cases, patients were not initially aware of the traumatic nature of these events. In this regard, the method of psychoanalysis, starting from what the patient spontaneously reports, may have an additional advantage over questionnaires providing a setting in which subjective and personally meaningful experiences can also be recognized as clinically relevant. Within this framework, the striking difference in the domain of social/family dysfunction between FND and controls suggests that not only overt abuse but also chronic relational difficulties may play a role in shaping vulnerability. This supports the need to broaden the focus beyond discrete traumatic events to include ongoing interpersonal stressors, which can be equally impactful yet less likely to be captured by standard tools.

Although the increased reporting of adverse events during psychotherapy could raise concerns related to memory suggestibility, our findings suggest that this phenomenon more likely reflects the emergence of previously unacknowledged or unrecognized experiences. Free-response formats may facilitate trauma reporting by enhancing awareness and meta-cognitive processing of how experiences are internalized and described. Patients with FND often show limited awareness of psychosocial or emotional contributors to symptom onset, which are frequently overlooked even during neurological evaluation, as historically noted by Freud and Breuer (37). Rather than implying fabrication or distortion, these dynamics may represent evolving interpretations of past events shaped by insight, emotional reprocessing, and the therapeutic alliance. This highlights the importance of considering the subjective dimension of trauma in FND and the clinical value of therapeutic dialogue in accessing emotionally salient material. Importantly, the psychotherapeutic intervention yielded a markedly higher detection rate in the FND group compared to the self-report questionnaire, whereas in the control group the increase was less pronounced. This significant method-by-group interaction reinforces the view that the increased reporting in FND patients cannot be entirely explained by a systematic reporting bias.

These findings also suggest that trauma-focused interventions may be clinically valuable in FND even when patients do not initially report traumatic stress, further supporting the need to explore such dimensions beyond standard screening.

Our study has some important limitations, including the relatively small sample size and the lack of follow up evaluations. In addition, our control group included heterogeneous neurological disorders. While this does not allow for direct symptom-matched comparisons, it provides a broad clinical reference group with comparable levels of depression and anxiety. Nevertheless, to our knowledge, this is the first study directly comparing standardized psychological/psychometric evaluation with a brief psychotherapeutic intervention for the assessment of traumatic/adverse life events in patients with FND. From this perspective, the psychotherapeutic approach appears to complement or even improve the results of standardized evaluations. Indeed, a program of 4–8 sessions may be demanding, costly and difficult to implement in many clinical settings. However, given the high prevalence, impact, and healthcare costs of FND, any strategy that improves detection of clinically relevant factors should be carefully considered. Because trauma is a well-recognized predisposing, precipitating, and perpetuating factor, having a more sensitive method to identify it may provide important clinical benefits.

When assessed through a personalized approach, such as the brief psychotherapeutic intervention, the prevalence of traumatic events in patients with FND might be higher than previously estimated. Psychosocial trauma, intrapsychic conflicts, and impaired emotion regulation are known to be common in these disorders and to contribute substantially to their clinical expression (38, 39). Our findings therefore suggest that psychotherapeutic assessment should be incorporated as an integral component of a multidisciplinary evaluation. While not challenging the current diagnostic approach, reaffirming a central role of trauma in the pathogenesis of FND may have possible implications for the treatment of these disorders.

## Data Availability

The raw data supporting the conclusions of this article will be made available by the authors, without undue reservation.
